# Less Grease, Please. Phosphatidylethanolamine Is the Only Lipid Required for Replication of a (+)RNA Virus

**DOI:** 10.3390/v7072784

**Published:** 2015-06-26

**Authors:** George A. Belov

**Affiliations:** Virginia–Maryland Regional College of Veterinary Medicine and Department of Veterinary Medicine, University of Maryland, College Park, MD 20742, USA; E-Mail: gbelov@umd.edu; Tel.: +1-301-314-1259

**Keywords:** +RNA virus replication, membranous replication organelles, membrane remodeling, phospholipids

## Abstract

All positive strand RNA viruses of eukaryotes replicate their genomes in association with membranes. These viruses actively change cellular lipid metabolism to build replication membranes enriched in specific lipids. The ubiquitous use of membranes by positive strand RNA viruses apparently holds major evolutionary advantages; however our understanding of the mechanistic role of membranes, let alone of specific lipid components of the membrane bilayer, in the viral replication cycle is minimal. The replication complexes that can be isolated from infected cells, or reconstituted *in vitro* from crude cell lysates, do not allow controlled manipulation of the membrane constituents thus limiting their usefulness for understanding how exactly membranes support the replication reaction. Recent work from Peter Nagy group demonstrates that replication of a model positive strand RNA virus can be reconstituted in the *in vitro* reaction with liposomes of chemically defined composition and reveals an exclusive role of phosphatidylethanolamine in sustaining efficient viral RNA replication. This study opens new possibilities for investigation of membrane contribution in the replication process that may ultimately lead to development of novel broad spectrum antiviral compounds targeting the membrane-dependent elements of the replication cycle conserved among diverse groups of viruses.

## 1. Introduction

Positive strand RNA ((+)RNA) viruses are arguably the most numerous viruses of eukaryotes [1]. They infect organisms from unicellular algae to the largest mammals and are often associated with economically and clinically important diseases of plants, animals, and humans. Genome size of these viruses is generally very small and, as the name implies, the information is encoded by an RNA molecule of mRNA polarity; in case of plant viruses the total genome is often split among two or more RNAs that need to enter the cell simultaneously for productive infection. Viral genome RNA(s) is directly translated by cellular ribosomes, thus they do not require previously synthesized viral proteins to start the infectious cycle, unlike many other more complex viruses. In spite of this deceptively simple if not primitive organization, these viruses can efficiently take control over the metabolism of highly complex eukaryotic cells. The scarcity of genetic resources apparently restricts their replication strategies, and these viruses rely on a few evolutionarily successful mechanisms targeting the basic metabolic processes conserved among eukaryotic cells. Indeed, on the cellular level the development of (+)RNA virus infection looks remarkably similar regardless of the host. Genome RNA replication of all (+)RNA viruses of eukaryotes is always associated with development of specialized membranous domains—replication organelles.

It is believed that membranes may promote recruitment of viral and cellular factors required for replication, facilitate proper topological assembly of multicomponent replication machinery, and hide dsRNA replication intermediates from cellular sensors of infection. However, the mechanistic details of how exactly the membranes are involved in the replication process remain elusive.

The main structural elements of biological membranes are phospholipids. These molecules consist of two functionally different parts. Two long chain fatty acid moieties attached to a glycerol backbone constitute the hydrophobic portion of the molecule and, when phospholipids form the lipid bilayer, the fatty acid composition determines membrane properties such as thickness, permeability, and fluidity, ultimately influencing membrane function as a biological barrier. The other part of a phospholipid molecule is a polar headgroup which faces the aqueous environment. Different headgroups, which possess different shapes and charges, determine specific recruitment and interactions of membrane-associated proteins and are an important part of membrane shaping mechanisms supporting positive (convex) or negative (concave) membrane curvature.

## 2. Phosphatidylethanolamine Supports Replication of a +RNA Virus

Xu and Nagy in their recent report in PNAS [2] provided a significant insight into the role of specific lipids in genome RNA replication of Tomato Bushy Stunt Virus (TBSV). Replication of TBSV and several other (+)RNA viruses of plants and insects can be studied in the yeast cells, unleashing the power of this model system for investigation of the role of host factors in viral replication. In case of TBSV, expression of only two viral proteins, p33 and p92, is sufficient to initiate formation of a fully functional replicase complex capable of replicating RNAs with the viral cis-acting replication signals [3]. p33 has RNA chaperon properties and is involved in RNA recruitment to the replication membranes and development of the replication compartments; p92 overlaps with p33 in its N-terminus and contains the C-terminal RNA-dependent RNA polymerase domain. During viral infection, p92 is synthesized via translational readthrough mechanism from the same RNA as p33, but in the yeast system expression of both proteins is driven by two different plasmids [3].

In this new study, Xu and Nagy could reconstitute TBSV replication *in vitro* with purified recombinant p33 and p92, membrane-free fraction of yeast cytosolic proteins, and artificial liposomes. By providing liposomes of different composition it was possible for the first time to directly investigate requirements of the viral replication reaction for specific phospholipids. Liposomes made from phosphatidylethanolamine (PE), a phospholipid with a positively charged conically shaped headgroup, supported replication of the template RNA up to half of the control level achieved when the system was supplemented with the membranous fraction from yeast cells. The requirement for PE was very specific, as liposomes prepared from lysophosphatidylethanolamine (LPE), a molecule similar to PE but without one of the fatty acid chains, supported only a barely detectable level of replication. The same background replication was detected with phosphatidylcholine (PC) liposomes, and no replication at all was observed when liposomes prepared from several other lipid species were tested. However, if the PE liposomes also contained about 10%–15% of a lipid mix mimicking the composition of intracellular membranes from *Nicotiana benthamiana*, a natural plant host for TBSV, the replication activity was significantly higher than that achieved with pure PE liposomes. The stimulatory effect could be partially recapitulated by addition of 10% of PC or LPE to PE liposomes, while at a concentration of more than 20% these lipids were inhibitory for replication.

The *in vitro* observations suggesting the important role of PE in TBSV replication were corroborated by several *in vivo* experiments. First, the authors showed that the total amount of PE is increased in yeast and *N. benthamiana* cells where TBSV RNA is replicating, and that PE content is significantly enriched at the replication sites. Second, replication of TBSV in yeast mutants with deletion of the PE methyltransferase gene resulting in accumulation of excess PE was up to 10 times more efficient than in wild type (wt) cells. Moreover, when the *in vitro* system with the same amount of recombinant TBSV proteins was reconstituted with the membrane fraction isolated from either wt or PE methyltransferase-deficient yeast, the latter could also support higher replication level of TBSV RNA replication. Finally, TBSV replication was significantly inhibited in yeast dependent on exogenous source of ethanolamine for PE synthesis when they were incubated in ethanolamine-free medium, and the replication could be rescued up to the wt level when ethanolamine was provided. Interestingly, while TBSV replication organelles normally develop on peroxisomal membranes, disruption of peroxisome biogenesis results in redistribution of the replication sites to the ER membranes [4]. Here Xu and Nagy showed that ER-associated TBSV replication sites are also enriched in PE, thus the virus can efficiently organize specific replication-promoting lipid environments regardless of the pre-existing composition of the cellular membrane. Moreover, similar enrichment of the replication sites in PE and correlation of the level of viral RNA replication with cellular PE synthesis were observed for cucumber necrosis virus and carnation Italian ringspot virus, two other tombusviruses related to TBSV, as well as for an unrelated insect Nodamura virus which can also replicate in yeast.

In principle, enrichment of the replication sites in PE could be achieved by localized *de novo* synthesis of the lipid and/or its active transport from the other cellular locations. Expression of only the p33 protein was sufficient to induce PE redistribution in the yeast cells. Since the exogenously added fluorescently labeled PE was found to be recruited to peroxisomal membranes in a p33-dependent manner, the active lipid transport must be involved in generation of the TBSV replication organelles, but the molecular mechanisms of how p33 controls the cellular lipid trafficking pathways remain to be elucidated.

How does specific lipid composition of the replication membranes, and in particular their enrichment in PE, stimulate the replication reaction? The TBSV replication sites *in vivo* represent membrane invaginations connected to the cytoplasm with narrow necks and the viral replication machinery is associated with the inner, negatively curved membrane surface. Enrichment of the membrane leaflet in PE with its conically shaped headgroup may promote development of the negative membrane curvature facilitating invagination development. Such spatial enclosure contributes to protection of the replicating RNA which becomes resistant to RNAse treatment [5]. However this mechanism is unlikely to play an important role in the reconstituted *in vitro* system since replicating RNA was easily degraded by micrococcal nuclease in the presence of PE liposomes. The membrane floatation assay also demonstrated that liposomes made from other phospholipids that were not effective in supporting replication could recruit viral replication proteins and template RNA just as good or even better than PE liposomes, excluding the possibility that high PE content is required simply for binding of the viral replication complexes to membranes. However, these experiments do not discriminate non-specific binding from assembly of the components of the replication machinery in the correct arrangement, leaving the possibility that PE promotes proper topological organization of the replication complex. Since the same amount of recombinant p33 and p92 and the yeast soluble protein fraction was added to the *in vitro* reactions containing different liposomes, it is possible that PE directly stimulates activity of a viral protein or a host factor required for replication. It was previously shown that phospholipids may stimulate specific steps in the replication cycle. Replication of (+)RNA viruses proceeds through synthesis of a −RNA strand on a genome RNA template, and the –strand further serves as a template for multiple rounds of synthesis of progeny +RNA strands. Exogenously added phospholipids activated productive synthesis of + strand RNAs, but were dispensable for the first −RNA strand synthesis by the isolated replication complexes of flockhouse virus, an insect (+)RNA virus [6]. In this case the replication reaction was stimulated by virtually any glycerophospholipid with PC being the most effective; thus different (+)RNA viruses may have different requirements for specific membrane composition. TBSV is a member of a so called flavi-like superfamily of (+)RNA viruses [7,8] that includes important human pathogens, such as Hepatitis C virus—which is estimated to infect more than 170 million people worldwide [9]; Dengue virus—one of the most important arthropod-borne viruses causing an estimated 390 million infections per year [10]; as well as West Nile virus—which can cause severe encephalitis and is rapidly spreading globally [11,12]. It would be interesting to see if these and other flavi-like viruses similarly require PE enrichment of the replication sites.

## 3. Concluding Remarks

The active manipulation of the cellular lipid synthesis and trafficking pathways by diverse (+)RNA viruses of plants and animals has been documented, showing that these viruses actively build their replication membranes with an optimal lipid microenvironment rather than just remodel pre-existing cellular organelles [13,14,15,16,17,18,19,20,21]. However, the critical questions of how viral proteins can override cellular control over lipid metabolism and what steps in the viral replication cycle actually require this specific membrane composition remain largely unanswered. The remarkable conservation of the replication strategies among (+)RNA viruses makes it very likely that even distantly related viruses rely on similar pathways to build their replication organelles. Elucidation of the molecular mechanisms engaged in their formation and functioning may ultimately reveal a whole new class of targets for development of effective broad spectrum antivirals.
